# Neural Network *L*_1_ Adaptive Control of MIMO Systems with Nonlinear Uncertainty

**DOI:** 10.1155/2014/942094

**Published:** 2014-03-30

**Authors:** Hong-tao Zhen, Xiao-hui Qi, Jie Li, Qing-min Tian

**Affiliations:** Department of UAV Engineering, Mechanical Engineering College, Shijiazhuang 050003, China

## Abstract

An indirect adaptive controller is developed for a class of multiple-input multiple-output (MIMO) nonlinear systems with unknown uncertainties. This control system is comprised of an *L*_1_ adaptive controller and an auxiliary neural network (NN) compensation controller. The *L*_1_ adaptive controller has guaranteed transient response in addition to stable tracking. In this architecture, a low-pass filter is adopted to guarantee fast adaptive rate without generating high-frequency oscillations in control signals. The auxiliary compensation controller is designed to approximate the unknown nonlinear functions by MIMO RBF neural networks to suppress the influence of uncertainties. NN weights are tuned on-line with no prior training and the project operator ensures the weights bounded. The global stability of the closed-system is derived based on the Lyapunov function. Numerical simulations of an MIMO system coupled with nonlinear uncertainties are used to illustrate the practical potential of our theoretical results.

## 1. Introduction

The control of nonlinear systems with uncertainties is still one of the hardest problems within control systems society. The following two factors make it difficult to design a high performance and yet universal enough controller for general uncertain nonlinear systems: (i) it is difficult to treat various nonlinearities under a unified framework, and (ii) in most situations, due to the limited knowledge about the system parameters or the external disturbance, it is also impossible to quantitatively describe the uncertainties.

In recent years, there has been a dramatic proliferation of research concerning the controller design for nonlinear systems. By many researchers, different control efforts have been developed from a point of view of adaptive control [1–5]. Khalil [1] addressed the adaptive tracking control of a class of nonlinear systems which can be represented by an input-output model. In [2], a modified adaptive backstepping design procedure was proposed for a broader class of nonlinear systems with a high degree of uncertainty. Hung et al. [3] developed a new adaptive control framework to compensate for uncertain nonlinear parameters in robot manipulators, and this controller can solve a very broad class of nonlinearly parameterized adaptive control problems and guarantee global boundedness of the closed-loop system.

Because of the coupling characteristic, the control design is very difficult for MIMO nonlinear systems and consequently the extension of the control design methods from SISO systems to MIMO systems is nontrivial in general. In recent decades, a quantity of work has been performed on MIMO systems with uncertainty. In [6], direct adaptive control was developed for a class of MIMO nonlinear systems in the presence of uncertain failures of redundant actuators. Chen et al. [7] proposed an adaptive tracking controller for a class of uncertain MIMO nonlinear systems with nonsymmetric input constraints; moreover, to avoid the tedious analytic computations of virtual control laws in the backstepping procedure, command filters were adopted to implement the emulate of actuator physical constraints.

Adaptive control has been widely used into various systems with uncertainties. Even so, adaptive control systems are designed by assuming that the plant is linear or by modeling the plant as a nonlinear system whose unknown parameters are linearly related to linear or nonlinear functions, and the adaptation law may lose stability even when a small disturbance appears [8]. In order to tackle the limitations of classical adaptive control systems, adaptive control of nonlinear systems with unknown functions has attracted increased interest. The most popular method is to incorporate feedback linearization method [9, 10], robust control technique [11–15], or intelligent system [16] into the adaptive control to attenuate the disturbance of unknown function. Feedback linearization adopts geometric technique to transform the certain nonlinear systems into a linear control problem, yet this scheme assumes that the plant should be described by known nonlinear functions precisely. Robust adaptive controller is composed of two components, in addition to the adaptive controller, and an additional robust item is adopted to attenuate the effect of the nonlinear function. However, although the asymptotic tracking is still preserved, the performance is conservative and the steady state tracking error can only be shown to stay within an unknown region, whose size depends on the disturbances. To approximate the unknown nonlinear function, two intelligent systems have been popular: fuzzy system [17–19] and neural network [20–26].

Starting from Narendra and Parthasarathy [20] firstly introducing the rigorous stability proof of neural network, the field has evolved significantly over the past two decades. Theoretically, as long as a sufficient number of neurons are employed, a neural network can approximate a continuous function to an arbitrary accuracy [21]. This universal approximation capability of neural network has enabled researchers to introduce it to control systems in the presence of general nonlinear uncertainties that could not be globally or linearly parameterized in unknown parameters. Hovakimyan et al. [22] proposed a Gaussian Radial Basis Neural Network using a tapped delay line of available measurement signals to compensate the modeling uncertainties for a class of nonminimum phase nonlinear systems. Rong et al. [23] presented an indirect adaptive neural control scheme based on the single-hidden layer feedforward network for a general high-order nonlinear continuous system.

Although a large amount of work has been carried out on the construction of adaptive controllers for SISO or MIMO nonlinear systems and that most of these works deduced the convergence performance of the tracking error, very few results mentioned the transient performance characterization. In practice, it is difficult to establish performance issues analytically on transient behavior (i.e., overshoot and convergence rate) even in the case of known nonlinearities. Such issues have been discussed only in terms of the *L*_2_ norm of the tracking error which is derived to be a function of explicit design parameters and initial estimation errors [27–29]. Lin et al. [30] proposed an output feedback variable structure model reference adaptive controller (VS-MRAC) with a high gain switching mechanism scheme for uncertain SISO linear plants to guarantee the prespecified transient performance specifications, but the involved infinite-gain feedback terms introduced control chattering. Bechlioulis and Rovithakis [31] presented two robust adaptive control schemes for SISO strict feedback nonlinear systems possessing unknown nonlinearities, capable of guaranteeing prescribed performance bounds. However, in these papers only the tracking errors were computed and the bounds of control signals were not considered.

In 2006, Cao and Hovakimyan [32, 33] firstly introduced the *L*_1_ adaptive control theory and applied this technique into various systems, such as state feedback and output feedback. The *L*_1_ adaptive control architecture hinges on an indirect architecture of model reference adaptive control (MRAC), which enables low-pass filtering of the control signal. The benefit of this new adaptive architecture is in its ability of fast adaptation that leads to desired transient response in addition to stable tracking for system's both signals input and output simultaneously.

In this paper, we extend the methodology from [34] to MIMO systems with unknown nonlinear function and define a neural network *L*_1_ adaptive controller. In comparison with the previous research, four main advantages of the proposed scheme can be summarized as follows. (a) Formulation of the plant is so general that it can represent various kinds of MIMO dynamic systems, and the coupling of the multiple variables has been taken into account. (b) A single-hidden-layer MIMO radial basis function (RBF) network is used to approximate the uncertain nonlinear functions. (c) Not only the stability of the closed-loop system is proved according to Lyapunov theory, but also the *L*_*∞*_ norms of tracking errors and control signals are deduced, which characterize the transient performance of input and output signals. (d) Bandwidth of the control channel can be chosen according to the performance of the actuator through the low-pass filter, but it does not influence the performance bounds obviously.

This paper is organized as follows.  Section 2 gives the problem formulation. In Section 3, the neural network *L*_1_ adaptive controller is presented. Stability and transient performance for the controller are analyzed in Section 4. In Section 5, simulation results are presented, while Section 6 concludes this paper.

Throughout this paper, the following notations are used:

- ||·|| stands for Euclidean norm of vectors and induced norm of matrices;
- *λ*_max⁡_(**B**) and *λ*_min⁡_(**B**) denote the largest and smallest eigenvalue of square matrix **B**, respectively;
- *χ*(*s*) denotes the Laplace transform of time signal *χ*(*t*).

## 2. Problem Formulation

In this paper, we are concerned with the following MIMO system dynamics:

(1) $$\begin{array}{c} \dot{x}\left(t\right)=Ax\left(t\right)+B\left(u\left(t\right)-f\left(t,x\left(t\right)\right)\right),\\ y\left(t\right)=Cx\left(t\right)\;\;x\left(0\right)=x_{0}, \end{array}$$

where **x** ∈ ℝ^*n*^ is the system state vector (measurable), **u** ∈ ℝ^*m*^ is the control signal (*m* ≤ *n*), **y** ∈ ℝ^*m*^ is the regulated output, **A** is a known *n* × *n* constant matrix, **B** is a known *n* × *m* constant matrix, and (**A**, **B**) is controllable, **C** ∈ ℝ^*m*×*n*^ is a known full-rank constant matrix, and (**A**, **C**) is observable; **f**(*t*, **x**) : ℝ × ℝ^*n*^ → ℝ^*m*^ is unknown nonlinear function which represents the general uncertainty.

Throughout this paper, we assume that the function **f**(**x**) satisfies the following conditions:

(A1) The function **f**(**x**) is Lipschitz continuous, so that there exists *L* such that
  (2) $$\begin{array}{c} {||f\left(x_{1}\right)-f\left(x_{2}\right)||}_{\infty }\leq L{||x_{1}-x_{2}||}_{\infty }. \end{array}$$
(A2) There exists *B* > 0 such that
  (3) $$\begin{array}{c} ||f\left(0\right)||\leq B \end{array}$$

holds for all *t* ≥ 0, where *B* is a known constant.

We further assume that the nonlinear function **f**(*t*, **x**(*t*)) can be approximated over a compact set *D*_*x*_ by an RBF neural network up to a desired accuracy [35]:

(4) $$\begin{array}{c} f\left(t,x\left(t\right)\right)=W^{\text{T}}\Phi \left(x\right)+\varepsilon \left(x\right), \end{array}$$

where **W** ∈ ℝ^*n*×*m*^ is a matrix of unknown parameters that belongs to a known (conservative) compact set **Ω** and, Φ(**x**) is a vector of Gaussian radial basis functions with its *i*th element:

(5) $$\begin{array}{c} \phi_{i}\left(x\right)=\exp\left(-\frac{{||x-z_{i}||}^{2}}{\delta_{i}^{2}}\right), \end{array}$$

where **z**_*i*_ and *δ*_*i*_ are the prefixed centers and widths, respectively, ||**ε**(**x**)||_*∞*_ ≤ ε* is the uniformly bounded approximation error, and ε* is a constant.

## 3. NN *L*_1_ Adaptive Control

In this section, we consider the problem of characterizing NN *L*_1_ adaptive full-state feedback control for nonlinear uncertain dynamical systems to achieve reference model trajectory tracking.

For system (1), consider the controller given by

(6) $$\begin{array}{c} u\left(t\right)=u_{b}\left(t\right)+u_{\text{ad}}\left(t\right), \end{array}$$

where **u**_*b*_(*t*) is the baseline controller:

(7) $$\begin{array}{c} u_{b}\left(t\right)=-K_{x}^{\text{T}}x\left(t\right)+K_{g}^{\text{T}}r\left(t\right), \end{array}$$

**u**_ad_(*t*) is the adaptive increment, **K**_*x*_ is a designed feedback gain matrix ensuring that **A**_*m*_ = **A** − **B** **K**_*x*_^T^ is Hurwitz, **K**_*g*_ = 1/**C** ^T^ **A**_*m*_^−1^ **B** ^T^ is the feedforward gain matrix that provides unit DC gains from the commanded signals to the corresponding system outputs, and **r**(*t*) is a bounded piecewise continuous reference input with known upper bound of ||**r**||_*L*_*∞*__.

Assuming no uncertainties (i.e., **f**(*t*, **x**(*t*)) = 0), the nominal controller **u**_*b*_(*t*) leads to the desired reference system

(8) $$\begin{array}{c} {\dot{x}}_{m}\left(t\right)=A_{m}x_{m}\left(t\right)+B_{m}r\left(t\right), \end{array}$$

where **x**_*m*_ ∈ ℝ^*n*^ is the reference state vector and **B**_*m*_ = **B** **K**_*g*_^T^.

The control objective is to design a state feedback controller to ensure that **y**(*t*) tracks the output response of desired system (8) both in transient and steady state, while all other error signals remain bounded. For this purpose, we design a neural network adaptive controller **u**_ad_(*t*) to cancel out the uncertainties. The complete controller (6) leads to the following closed-loop dynamics:

(9) $$\begin{array}{c} \dot{x}\left(t\right)=A_{m}x\left(t\right)+B_{m}r\left(t\right)+B\left(u_{\text{ad}}\left(t\right)-f\left(t,x\left(t\right)\right)\right),\\ y\left(t\right)=Cx\left(t\right)\;\;x\left(0\right)=x_{0}. \end{array}$$

Substituting the RBF NN (4) into (9) leads to the linearly parameterized system dynamics:

(10) $$\begin{array}{c} \dot{x}\left(t\right)=A_{m}x\left(t\right)+B_{m}r\left(t\right)+B\left(u_{\text{ad}}\left(t\right)-W^{\text{T}}\left(t\right)\Phi \left(x\right)-\varepsilon \left(x\right)\right). \end{array}$$

For system (10), we consider the following state predictor:

(11) $$\begin{array}{c} \dot{\hat{x}}\left(t\right)=A_{m}\hat{x}\left(t\right)+B_{m}r\left(t\right)+B\left(u_{\text{ad}}\left(t\right)-{\hat{W}}^{\text{T}}\left(t\right)\Phi \left(x\right)\right),\\ \hat{y}\left(t\right)=C\hat{x}\left(t\right)\;\;\hat{x}\left(0\right)=x_{0}, \end{array}$$

where $\hat{x}\in {\mathbb{R}}^{n}$ is the prediction state vector and $\hat{W}(t)$ is the adaptive parameter. Then, the following error dynamics can be derived from (9) and (11):

(12) $$\begin{array}{c} \dot{\tilde{x}}\left(t\right)=A_{m}\tilde{x}\left(t\right)-B{\tilde{W}}^{\text{T}}\left(t\right)\Phi \left(x\right)+B\varepsilon \left(x\right),\;\;\tilde{x}\left(0\right)=0, \end{array}$$

where $\tilde{x}(t)=\hat{x}(t)\;-\;x(t)$ is tracking error and $\tilde{W}\left(t\right)=\hat{W}\left(t\right)-W\left(t\right)$ is estimate error.

Define the adaptive laws as follows:

(13) $$\begin{array}{c} \dot{\hat{W}}\left(t\right)=\Gamma \text{Proj}\left(\hat{W}\left(t\right),\Phi \left(x\right)\tilde{x}PB\right), \end{array}$$

where Γ  is the positive adaptation gain, **P** = **P** ^T^ > 0 is the solution of the algebraic Lyapunov equation **A**_*m*_^T^ **P** + **P** **A**_*m*_ = −**Q** for arbitrary symmetric **Q** = **Q** ^T^ > 0, and Proj(·, ·) denotes the projection operator [36]

(14) Proj(θ,y)≜{yif f(θ)<0,yif f(θ)≥0 , ∇fTy≤0,y−∇f||∇f||〈∇f||∇f||,y〉f(θ)if f(θ)≥0, ∇fTy>0,

where *f* is the following smooth convex function:

(15) f(θ)≜(εθ+1)θTθ−θmax⁡2εθθmax⁡2,

with *θ*_max⁡_ being the norm bound imposed on the vector ***θ***, and *ε*_*θ*_ > 0 is the projection tolerance bound of our choice.

Letting

(16) $$\begin{array}{c} \overset{-}{r}\left(t\right)={\hat{W}}^{\text{T}}\left(t\right)\Phi \left(x\right), \end{array}$$

then the adaptive controller can be designed as

(17) $$\begin{array}{c} u_{\text{ad}}\left(s\right)=C\left(s\right)\overset{-}{r}\left(s\right), \end{array}$$

where **C**(*s*) is a diagonal transfer function matrix with *C*_*i*_(*s*) strictly proper stable and low-pass gain *C*_*i*_(0) = 1 and $\overset{-}{r}(s)$ is the Laplace transformation of $\overset{-}{r}(t)$.

**Remark 1**

Considering the Laplace transform of system (11) with the controller defined in (17) as follows:

(18) $$\begin{array}{c} \hat{x}\left(s\right)=G\left(s\right)r\left(s\right)+\overset{-}{G}\left(s\right)\overset{-}{r}\left(s\right), \end{array}$$

(19) $$\begin{array}{c} G\left(s\right)={\left(sI-A_{m}\right)}^{-1}B_{m}, \end{array}$$

(20) $$\begin{array}{c} \overset{-}{G}\left(s\right)={\left(sI-A_{m}\right)}^{-1}B\left(C\left(s\right)-I\right), \end{array}$$

it can be viewed as an LTI system with two inputs, reference input signal **r**(*t*), and time-varying disturbance $\overset{-}{r}(t)$ which is related to **f**(**x**). **G**(*s*) is the transfer function of the desired reference system (8) and $\overset{-}{G}(s)$ can be viewed as the transfer function of $\overset{-}{r}(t)$.

**Remark 2**

Equation (20) implies that $\overset{-}{G}(s)$ can be viewed as the cascade of a low-pass system:

(21) $$\begin{array}{c} H\left(s\right)={\left(sI-A_{m}\right)}^{-1}B \end{array}$$

and a high-pass system (**C**(*s*) − **I**). Then, if the bandwidth of **C**(*s*), which approximately corresponds to the cut-off frequency of (**C**(*s*) − **I**), is designed to be larger than the bandwidth of **H**(*s*), the resulting $\overset{-}{G}(s)$ will be a “no-pass filter.” So, to ensure that the close-loop system (10) tracks the desired reference system (8), the design of **C**(*s*) has to satisfy the *L*_1_ gain requirement:

(22) $$\begin{array}{c} {||\overset{-}{G}\left(s\right)||}_{L_{1}}<\frac{1}{L}, \end{array}$$

where the *L*_1_ norm definition can be found in [37]. The illustration of requirement (22) will be discussed in Section 4.

**Figure 1:**
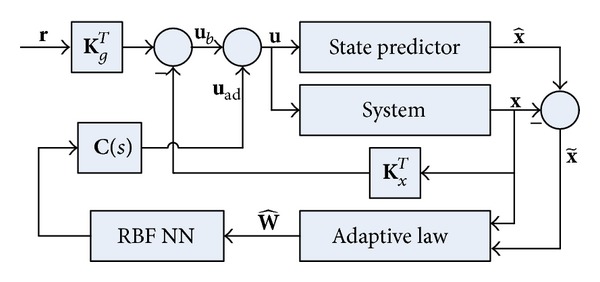
Closed-loop system with NN *L*_1_ adaptive controller.

The complete neural network *L*_1_ adaptive controller consists of (6), (7), (11), (13), and (17) subject to (22). The closed-loop system architecture is presented in Figure 1.

## 4. Analysis of NN *L*_1_ Adaptive Controller

### 4.1. Stability Analysis

Considering error dynamics (12), the following lemma will state that the system is stable and its state is bounded.

**Lemma 3**

Consider that the closed-loop system consists of error dynamics (12) and adaptive law (13); all of the signals in this system are uniformly bounded and the tracking error is as follows:

(23) $$\begin{array}{c} {||\tilde{x}||}_{L_{\infty }}\leq \frac{2||PB_{m}||\varepsilon^{*}}{\lambda_{\min}\left(Q\right)}, \end{array}$$

where *λ*_min⁡_(**Q**) is the minimum eigenvalue of **Q**; **P** and **Q** are introduced in (13).

**Proof**

The proof is given in the Appendix.

**Remark 4**

We notice that the bound in (23) is derived independently of **u**_ad_(*t*), and this implies that we cannot apply Lyapunov theory or Barbalat's lemma to conclude asymptotic convergence of **x**(*t*). Both $\hat{x}(t)$ and **x**(*t*) can diverge at the same rate, maintaining a uniformly bounded error of $\tilde{x}(t)$.

Next, we will prove that, with the adaptive feedback (17), the prediction state $\hat{x}(t)$ remains bounded and consequently leads to asymptotic stable of closed-loop system (9).

**Lemma 5**

If **u**_*ad*_ is defined as (17) and condition (22) holds, the prediction state $\hat{x}(t)$ will be uniformly bounded:

(24) ||x^(t)||L∞≤||G(s)||L1||r(t)||L∞1−λ+||G−(s)||L1(L0+ε∗)1−λ +λ(2||PBm||ε∗/λmin⁡(Q))1−λ,

where

(25) $$\begin{array}{c} \lambda ={||\overset{-}{G}\left(s\right)||}_{L_{1}}L. \end{array}$$

**Proof**

The proof is given in the Appendix.

**Theorem 6**

For system (1) and controller defined via (7) and (17) subject to the *L*_1_ gain requirement (22), the closed-loop system (9) is stable.

**Proof**

Using Lemmas 3 and 5, we immediately conclude that $\tilde{x}(t)$ and $\hat{x}(t)$ are bounded. At the same time, the adaptive laws in (13) ensure that $\hat{W}(t)$ is bounded. Hence, it can be checked straightforwardly that all of the signals in the closed-loop system (9) are bounded; that is to say, the system is stable. This completes the proof of Theorem 6.

### 4.2. Transient Performance Analysis

From the relationships ((A.8)), we have

(26) ||x(t)||L∞≤||G(s)||L1||r(t)||L∞1−λ+||G−(s)||L1(L0+ε∗)1−λ +λ(2||PBm||ε∗/λmin⁡(Q))1−λ +2||PBm||ε∗λmin⁡(Q)

which states that **x**(*t*) is uniformly bounded including the transient phase, as long as the NN approximation is accurate enough.

Furthermore, considering the expressions (6), (7), and (17), they lead to the following relationship:

(27) $$\begin{array}{c} u\left(s\right)=-K_{x}^{\text{T}}x\left(s\right)+K_{g}^{\text{T}}r\left(s\right)+C\left(s\right)\overset{-}{r}\left(s\right). \end{array}$$

Consequently, the following bound holds:

(28) ||u(s)||L∞≤||KxT||L1||x(t)||L∞+||KgT||L1||r(t)||L∞ +||C(t)||L1||r−(t)||L∞.

Substituting (23), (24), ((A.9)), and (26) into (27) yields

(29) ||u(s)||L∞≤(||KxT||L1+||C(t)||L1L)ρ+||KgT||L1||r(t)||L∞ +||C(t)||L1(L0+ε∗),

where

(30) ρ=||G(s)||L1||r(t)||L∞1−λ+||G−(s)||L1(L0+ε∗)1−λ +λ(2||PBm||ε∗/λmin⁡(Q))1−λ +2||PBm||ε∗λmin⁡(Q).

Equation (29) illustrates that the control signals of neural network *L*_1_ adaptive control architecture are uniformly bounded, as long as we choose proper **K**_*x*_, **K**_*g*_, and neural network. This performance is very useful for the problem of actuator saturation constrain.

**Remark 7**

From the analysis above, it follows that the *L*_1_ adaptive controller can generate a system response to track (8) both in transient and steady state if we set the adaptive gain to be large and minimize ${||\overset{-}{G}(s)||}_{L_{1}}$. Notice that **u**(*t*) depends upon the RBF approximation $\overset{-}{r}\left(t\right)$, and this implies that for different nonlinearities **f**(**x**), the neural network *L*_1_ adaptive controller will generate different control signal to ensure uniform system response. It also implies that the tracking accuracy depends on the estimating performance of RBF.

## 5. Numerical Simulations

In this section, by using the method of steps for differential equations, we give some numerical simulations to illustrate the theoretical results above.

Consider the following MIMO system with nonlinear uncertainties:

(31) $$\begin{array}{c} \dot{x}\left(t\right)=Ax\left(t\right)+B\left(u\left(t\right)-f\left(t,x\left(t\right)\right)\right),\\ y\left(t\right)=Cx\left(t\right)\;\;x\left(0\right)=x_{0}, \end{array}$$

where

(32) A=[−1000−3000−2],  B=[100010002],C=[100010001],

**x** = [*x*_1_ *x*_2_ *x*_3_]^T^ ∈ ℝ^3^ is the measurable state vector, the initial state **x**_0_ = [0 0 0]^T^, **u** ∈ ℝ^3^ is the control signal, **y**(*t*) ∈ ℝ^3^ is the output signal, and **f**(**x**) is an unknown nonlinear function of system states. The control objective is to design an NN *L*_1_ adaptive controller **u**(*t*) to ensure that the output of the system **y**(*t*) tracks the output of the desired system **G**(*s*) for bounded reference inputs signal **r**(*t*), both in transient and steady state phases. In following simulations, we consider the uncertainties:

(33) $$\begin{array}{c} f\left(x\right)=\left[\begin{array}{c} x_{1}x_{2}\\ 0.5+\text{0.3tanh}\left(x_{3}\right)\\ 0.3x_{3}^{2} \end{array}\right]. \end{array}$$

For the *L*_1_ adaptive controller, we set

(34) $$\begin{array}{c} K_{x}=0_{3\times 3},\;\;A_{m}=A,\;\;Q=I_{3\times 3},\\ \Gamma =500,\;\;L=1,\\ {||\overset{-}{G}\left(s\right)||}_{L_{1}}L=0.6528,\;\;C_{i}\left(s\right)=\frac{3\omega^{2}s+\omega^{3}}{{\left(s+\omega \right)}^{3}},\;\omega =5,\\ P=\left[\begin{array}{ccc} 0.50 & 0 & 0\\ 0 & 0.17 & 0\\ 0 & 0 & 0.25 \end{array}\right],\;\;K_{g}=\left[\begin{array}{ccc} 1 & 0 & 0\\ 0 & 3 & 0\\ 0 & 0 & 1 \end{array}\right]. \end{array}$$

**Figure 2:**
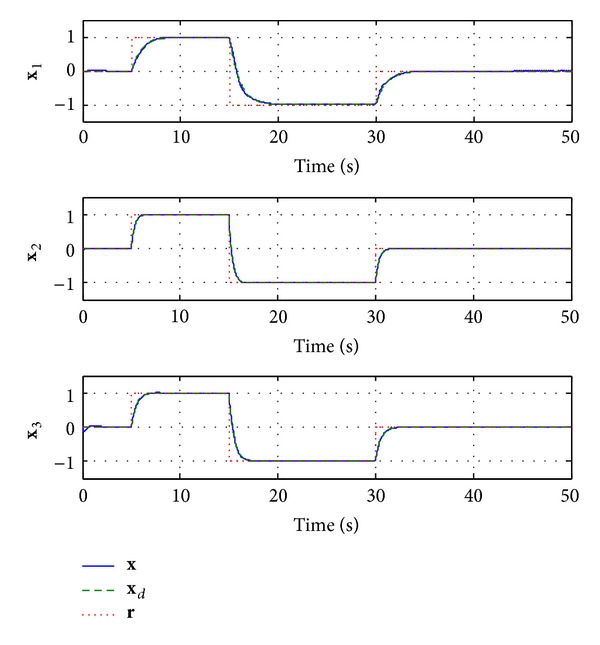
Response of the system with the nonlinear uncertainties.

**Figure 3:**
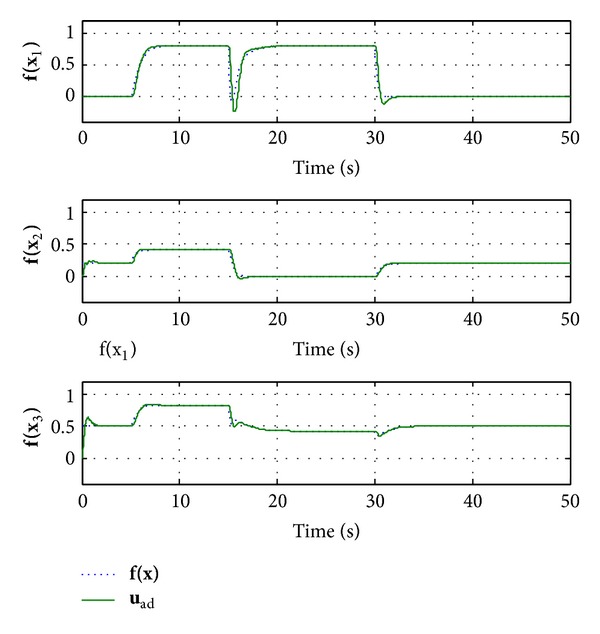
Nonlinear function **f**(**x**) and adaptive increment **u**_ad_.

**Figure 4:**
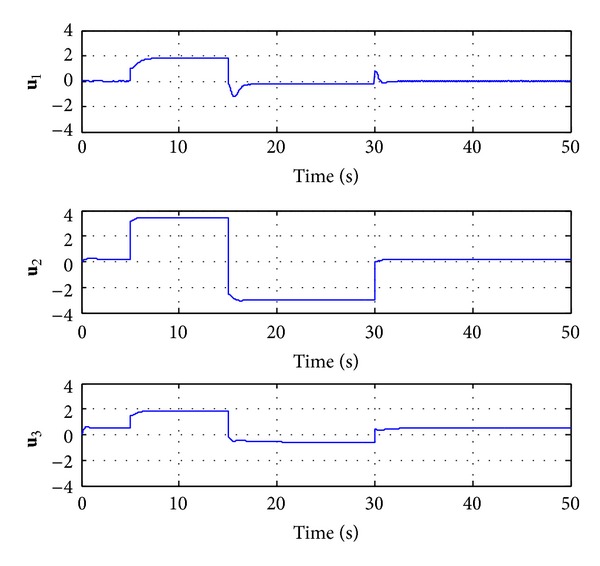
Time history of **u**(*t*).

**Figure 5:**
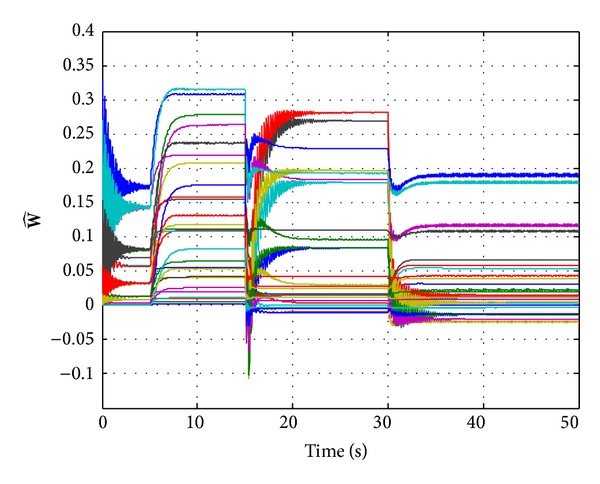
Evolution of weights $\hat{W}$.

The hidden layer of the RBF neural network includes 9 neurons, and the prefixed center **z**_*i*_ is distributed in [−2,2] with the increment 0.5 and width *δ*_*i*_ = 2. The simulation results are shown in Figures 2–5.

Figure 2 depicts the response of the closed-loop system to a series of step reference inputs with different amplitudes. The solid line **x** represents the actual outputs of the closed-system, the dashed line **x**_*d*_ represents the outputs of the desired reference system (8), and the dotted line **r** represents the reference inputs. One can observe that the neural network *L*_1_ adaptive controller guarantees smooth transient performance and uniform steady state performance in the presence of nonlinear uncertainties. Moreover, the response of the closed-loop system is close to the desired system, whose performance specifications are desired.

**Figure 6:**
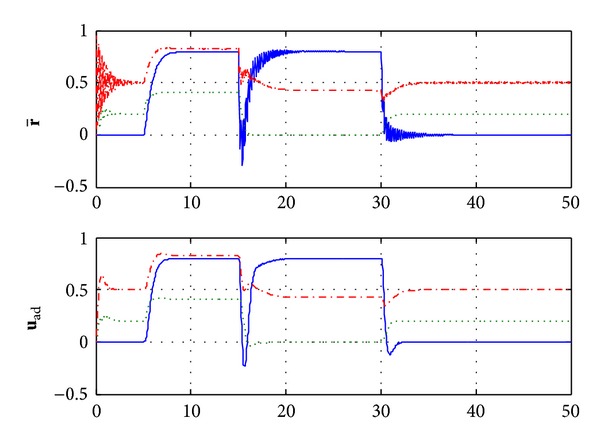
NN approximation $\overset{-}{r}$ and adaptive increment **u**_ad_.

From Figure 3, we can note that the incremental adaptive controller compensates for the unknown disturbance completely and leads to desired response. Furthermore, Figure 4 illustrates that the control signal in each channel is bounded.  Figure 5 shows the estimated values of the 27 weights in the RBF neural networks. Due to the large adaptive gain, one can see some unexpected oscillations in this figure. Consequently, this leads to the chattering in neural networks approximation $\overset{-}{r}$ of nonlinear function **f**(**x**), as shown on the top half of Figure 6. In order to compensate the effects of the unknown function, the adaptive control signals have to duplicate the oscillations, yet this will hurt the transient performance, and it will be difficult to be implemented in reality. The low-pass filter **C**(*s*) abates the chattering but does not hurt the control performance significantly, as it is illustrated on the bottom half of Figure 6. Furthermore, the bandwidth of **C**(*s*) can be determined according to the performance of actuator.

## 6. Conclusion

Neural networks and *L*_1_ adaptive control design philosophy have been integrated to design a controller for a class of nonlinear MIMO systems with unknown uncertainties. The unknown nonlinear functions are approximated by an MIMO RBF neural network to achieve a better model compensation. NN weights are tuned on-line with no prior training needed. The *L*_1_ adaptive controller has guaranteed transient response in addition to stable tracking. The low-pass filter guarantees fast adaptive rate without high-frequency oscillations in the control signal. Simulation studies on a nonlinear MIMO system were clarified and verified the proposed approach.

## Appendix

Proof of Lemma 3
Choose the following Lyapunov function candidate:

(A.1) $$\begin{array}{c} V\left(\tilde{x}\left(t\right),\tilde{W}\left(t\right)\right)=\tilde{x}{\left(t\right)}^{\text{T}}P\tilde{x}\left(t\right)+\frac{1}{\Gamma }\mathrm{tr}\left({\tilde{W}}^{\text{T}}\left(t\right)\tilde{W}\left(t\right)\right). \end{array}$$

It is obvious that the function is positive definite. The time derivative of *V* is given by

(A.2) V˙(t)=x~˙TPx~+x~TPx~˙+2Γtr⁡(W~TW~˙)=x~T(AmP+PAm)x~−2x~TPBmW~TΦ +2x~TPBmε+2Γtr⁡(W~TW~˙)=−x~TQx~−2x~TPBmW~TΦ+2x~TPBmε +2tr⁡(W~TProj(W^,Φx~TPBm))=−x~TQx~+2tr⁡(W~T(Proj(W^,Φx~TPBm)−  Φx~TPBm))+2x~TPBmε≤−x~TQx~+2PBmε≤−x~TQx~+2||x~T||||PBm||||ε||≤−λmin⁡(Q)||x~T||2+2||x~T||||PBm||ε∗=−||x~T||(λmin⁡(Q)||x~T||−||PBm||ε∗).

Therefore, $\dot{V}(t)\leq 0$ if

(A.3) $$\begin{array}{c} ||\tilde{x}||\geq \frac{2||PB_{m}||\varepsilon^{*}}{\lambda_{\min}\left(Q\right)}, \end{array}$$

which implies that $\tilde{x}(t)$ is uniformly bounded. It follows from (A.2), (A.3), and the fact ||·||_*∞*_ ≤ ||·|| that

(A.4) $$\begin{array}{c} {||\tilde{x}||}_{L_{\infty }}\leq \frac{2||PB_{m}||\varepsilon^{*}}{\lambda_{\min}\left(Q\right)}. \end{array}$$

Furthermore, the projection algorithm ensures $\hat{W}(t)\in \Omega$, so all of the signals in this system are bounded.

Proof of Lemma 5
Considering the closed-loop state predictor (11), (13), and (17), according to Lemma 1 in [34] leads to the following upper bound:

(A.5) $$\begin{array}{c} {||\hat{x}\left(t\right)||}_{L_{\infty }}\leq {||G\left(s\right)||}_{L_{1}}{||r\left(t\right)||}_{L_{\infty }}+{||\overset{-}{G}\left(s\right)||}_{L_{1}}{||\overset{-}{r}\left(t\right)||}_{L_{\infty }}. \end{array}$$

Applying the triangular relationship for norms

(A.6) $$\begin{array}{c} \left|||a||-||b||\right|\leq ||a-b|| \end{array}$$

to the bound (A.4), we have

(A.7) $$\begin{array}{c} \left|{||\hat{x}\left(t\right)||}_{L_{\infty }}-{||x\left(t\right)||}_{L_{\infty }}\right|\leq \frac{2||PB_{m}||\varepsilon^{*}}{\lambda_{\min}\left(Q\right)}. \end{array}$$

From the resolution geometry, we have

(A.8) $$\begin{array}{c} {||x\left(t\right)||}_{L_{\infty }}\leq {||\hat{x}\left(t\right)||}_{L_{\infty }}+\frac{2||PB_{m}||\varepsilon^{*}}{\lambda_{\min}\left(Q\right)}. \end{array}$$

From the definition of **f**(**x**) in (4) and $\overset{-}{r}(t)$ in (16), we have

(A.9) ||r−(t)||L∞=f(x)−ε(x)≤|f(0)|+L||x(t)||∞+ε∗≤L0+L(||x^(t)||L∞+2||PBm||ε∗λmin⁡(Q))+ε∗.

Substituting (A.9) into (A.5) yields

(A.10) ||x^(t)||L∞≤||G(s)||L1||r(t)||L∞+||G−(s)||L1 ×(L0+L(||x^(t)||L∞+2||PBm||ε∗λmin⁡(Q))+ε∗)=||G(s)||L1||r(t)||L∞+||G−(s)||L1L||x^(t)||L∞ +||G−(s)||L1(L0+ε∗)+||G−(s)||L1L2||PBm||ε∗λmin⁡(Q).

From the *L*_1_ gain requirement (22), we have

(A.11) ||x^(t)||L∞≤||G(s)||L1||r(t)||L∞1−λ+||G−(s)||L1(L0+ε∗)1−λ+λ(2||PBm||ε∗/λmin⁡(Q))1−λ.

Since the bound on the right-hand side is uniform, $\hat{x}(t)$ is uniformly bounded and ${||\hat{x}(t)||}_{L_{\infty }}\leq \rho$.
